# Delegates to the American Medical Association

**Published:** 1867-07

**Authors:** N. S. Davis, P. H. Bailhache

**Affiliations:** Permanent Sec’y.; Ass’t-Sec’y.


On motion of Dr. D. W. Young, the Society proceeded to elect the following members as delegates to the next annual meeting of the American Medical Association:—

Drs. T. F. Worrell, of Bloomington.
J. H. Hollister, of Chicago.
C. Goodbrake, of Clinton.
Jos. Robbins, of Quincy.
W. S. Edgar, of Jacksonville.
H. B. Buck, of Springfield.
F. B. Haller, of Vandalia.
Moses Gunn, of Chicago.
J. C. Corbus, of LaSalle.
J. T. Wilson, of Quincy.
H. Noble, of Heyworth.
N. Wright, of Chatham.
L. T. Hewins, of Loda.
H. W. Davis, of Paris.
B. K. Shurtleff, of Amboy.
J. M. Steele of Grandview.
E. H. Gale, of Aurora.

Dr. P. H. Bailhache reported that the Treasurer's report has been examined by the Auditing Committee, and found correct.

Dr. J. S. Hildreth offered the following, which was referred to the Committee on Legislation:—

*Resolved*, That a committee of three be appointed, to report at the next annual meeting of this Society, upon the necessity of an act of the Legislature defining the duties, responsibilities, and liabilities of Druggists and Pharmaceutists.

On motion, the Society adjourned, until 7 o'clock P.M.

EVENING SESSION—SECOND DAY.

The Society was called to order by the President at 7 o'clock P.M.

Dr. F. O. Earle, of Chicago, read a paper on the Mechanical Treatment of Angular Curvature of the Spine, and exhibited apparatus. The paper was accepted and referred to the Committee on Publication.

Dr. Addison Niles presented a specimen of Pocket Midwifery Forceps, with a description of the same, which was referred to the Committee of Publication.

Dr. Higgins, of Vandalia, presented the history of a Case of Hydrophobia, which was read by Dr. F. B. Haller, and elicited some discussion. The thanks of the Society were tendered to Dr. Higgins, with the request that the case be published in some medical journal.

Dr. H. A. Johnson offered the following resolution, which was adopted:—

*Resolved*, That this Society urge upon the municipal authorities of all our cities and larger towns the importance of a careful record of births, and a uniform registration of deaths and their causes, using for this purpose such necrological tables as have been generally adopted in this country and Europe.

Dr. D. Prince offered the following, which was adopted:—

*Resolved*, That the Committee on Legislation be instructed to consider the propriety of urging upon the Legislature the passage of a law requiring railroad companies, and other incorporated companies using machinery, to be responsible for the expense of board, nursing, medical supplies, and medical attendance necessary for their employees during the process of recovery, not exceeding six months, in cases of injuries received in the performance of their duties.

The resolution relating to medical education, previously offered by Dr. F. B. Haller, and deferred until the reports of committees, etc., had been disposed of, was now taken up, and after some remarks by Dr. Haller, it was, on motion of Dr. J. S. Whitmire, referred to a committee of three, to report on the same at the next annual meeting of the Society. Drs. F. B. Haller, S. T. Trowbridge, and H. Noble were appointed said committee.

The President also appointed the following committee to examine and report on the status of the Quincy Medical Society: Drs. C. Goodbrake, E. L. Holmes, and D. O. Crist.

The following resolutions were unanimously adopted:—

*Resolved*, That the thanks of this Society be and are hereby tendered to the retiring officers, for their faithful performance of their respective duties.

*Resolved*, That the thanks of this Society are hereby tendered to the profession, citizens, and authorities of Springfield, and also to the Committee of Arrangements, for the provision made for the accommodation and entertainment of the Society at this meeting.

*Resolved*, That the thanks of the Society be tendered to Hon. Sharon Tyndale, the Hon. Secretary of State, for his courtesy in granting the use of the Hall of Representatives for the sessions of the State Medical Society.

*Resolved*, That the thanks of the Society be tendered to the Superintendents of the Illinois Central R.R., the Chicago, Alton and St. Louis R.R., and the Toledo, Wabash, and Great Western R.R., for their favors of commutation tickets to members of the Society attending this meeting.

On motion, the Society adjourned *sine die*.

N. S. DAVIS, *Permanent Sec'y.*
P. H. BAILHACHE, *Ass't-Sec'y.*

